# MdMYC2 and MdERF3 Positively Co-Regulate α-Farnesene Biosynthesis in Apple

**DOI:** 10.3389/fpls.2020.512844

**Published:** 2020-09-02

**Authors:** Qing Wang, Heng Liu, Min Zhang, Shaohua Liu, Yujin Hao, Yuanhu Zhang

**Affiliations:** ^1^ State Key Laboratory of Crop Biology, College of Life Sciences, Shandong Agricultural University, Tai’an, China; ^2^ College of Horticulture Science and Engineering, Shandong Agricultural University, Tai’an, China

**Keywords:** apple, α-farnesene, α-farnesene synthase, transcriptional regulation, MdMYC2, MdERF3, genetic engineering, terpenes biosynthesis

## Abstract

α-Farnesene, a sesquiterpene volatile compound plays an important role in plant defense and is known to be associated with insect attraction and with superficial scald of apple and pear fruits during cold storage. But the mechanism whereby transcription factors regulate apple α-farnesene biosynthesis has not been clarified. Here, we report that two transcription factors, MdMYC2 and MdERF3 regulated α-farnesene biosynthesis in apple fruit. Dual-luciferase assays and Y1H assays indicated that MdMYC2 and MdERF3 effectively trans-activated the *MdAFS* promoter. EMSAs showed that MdERF3 directly binds the DRE motif in the *MdAFS* promoter. Subsequently, overexpression of *MdMYC2* and *MdERF3* in apple calli markedly activated the transcript levels of *MdHMGR2* and *MdAFS*. Furthermore, transient overexpression of *MdMYC2* and *MdERF3* in apple fruit significantly increased *MdAFS* expression and hence, α-farnesene production. These results indicate that MdMYC2 and MdERF3 are positive regulators of α-farnesene biosynthesis and have important value in genetic engineering of α-farnesene production.

## Introduction

Plants produce a large number of metabolites that are essential for ecological interactions and terpenes are natural products of the largest and structurally most diverse class (Reddy et al., 2017). Terpenes play an important role in the communication between plants and the environment, between plants and animals, and between plants and plants; they can attract pollinators and seed spreaders, and they can act as defense agents against herbivores and pathogens. In addition, they protect plants from abiotic stress phenomena, such as high light intensity, high temperature, and oxidative stress, among others. Terpenes are also useful nutrients in human diet and are used as chemotherapeutic agents for their antitumor activities (Dudareva et al., 2006).

α-Farnesene is an abundant compound in apple peel, which has an important function in plant defense (Nieuwenhuizen et al., 2014). The oxidative product of α-farnesene, are widely considered as a principal cause of scald, the physiological disorder of apple and pear, which intensify when fruits are transferred to ambient temperature for transportation to the market (Huelin and Coggiola, 1970; Ingle and D’Souza, 1989; Rowan et al., 1995). Overexpression of α-farnesene synthase in pathogen-sensitive soybeans enhances plant antimicrobial activity and α-farnesene has also been considered as a potential biofuel precursor (Lin et al., 2017).

There are three kinds enzymes involved in α-farnesene metabolism: 1) 3-hydroxy-3-methylglutaryl-CoA reductase (HMGR), which initiates synthesize sesquiterpenes (Rupasinghe et al., 2001; Zhang et al., 2020); 2) farnesyl diphosphate synthase (FPS), which catalyzes the conversion of isopentenyl pyrophosphate (IPP) and dimethylallyl pyrophosphate (DMAPP) to farnesyl diphosphate (FPP), the substrate of α-farnesene synthesis; 3) α-farnesene synthase (AFS), which catalyzes the final rate-limiting step in α-farnesene biosynthesis (Pechous and Whitaker, 2004; Gapper et al., 2006); But in plant the regulatory mechanisms of α-farnesene biosynthesis remains largely unknown.

The synthesis of terpenes in plants is regulated by many transcription factors, including MYC, AP2/ERF, bZIP, WRKY. MYC2 is important for JA response to secondary metabolite accumulation. *AtMYC2* binds the promoters of *AtTPS11* and *AtTPS21* for regulating the synthesis of sesquiterpenes in *Arabidopsis thaliana* (Hong et al., 2012). In *Artemisia annua*, *AaMYC2* binds the G-boxlike motifs within the promoters of genes *CYP71AV1* and *DBR2* (Shen et al., 2016). In *Salvia miltiorrhiza*, *SmMYC2a* and *SmMYC2b* play important roles in regulating the biosynthesis of phenolic acids (Zhou et al., 2016). Ethylene response factors have also been well characterized for their roles in regulating the production of terpenes. *AaERF1* and *AaERF2* bound to the promoter of *AaADS* for inducing artemisinin synthesis (Yu et al., 2012). *TcERF15*, respectively, act as activator of tasy gene of taxol biosynthesis in *Taxus chinensis* (Zhang et al., 2015). In Newhall sweet orange fruit, the transcription factor *CitERF71* directly binds the *CitTPS16* promoter, therefore probably has a function in the transcriptional regulation of *E*-geraniol production (Li et al., 2017).

Although transcription factors related to the metabolic pathway of terpenes have been reported in recent years, there are few reports on transcription factors related to the synthesis of terpenes in apples. At present, regardless of the metabolic engineering research of α-farnesene or the biological research of apple superficial scald, transcription factors regulating the mechanism of α-farnesene biosynthesis have not been reported. By screening transcription factors involved in secondary metabolic regulation, we studied whether these transcription factors are participated in regulating the expression of α-farnesene synthase, thereby affecting the biosynthesis of α-farnesene.

Our results revealed that transcription factors MdMYC2 and MdERF3 effectively activated the promoter region of *MdAFS*, which is the terminal enzyme gene in the α-farnesene biosynthesis pathway; additionally, they activated the expression of the *MdAFS* gene, and ultimately promoted the accumulation the α-farnesene.

## Materials and Methods

### Plant Materials and Treatments

Leaves obtained from five-year-old apple (Malus domestica Borkh. cv. White winter pearmain) trees were used in this study. The apple trees were cultivated in a culture room at Shandong Agriculture University. The “Orin” apple calli were used for genetic transformation and were grown at 24°C under dark conditions.

“White winter pearmains” apple leaves were treated with 100 μM MeJA and 50 mg L^-1^ Ethephon (Sigma-Aldrich), with 0.1% (v/v) ethanol as the mock. Samples were taken after 0, 2, 4, 6, and 12 h to analyze gene expression.

Fruits harvested at 140 days after full bloom were divided into four groups. The first group was not treated. The second group was treated with methyl jasmonate for 5 min. The third group was used for Ethephon treatments for 30 s. The fourth group was treated with 1-MCP for 12 h. All fruits were stored at room temperature (24°C) and 0°C for 4 weeks, with sampling every week during the storage period. The samples were frozen immediately in liquid nitrogen and then stored at -80°C.

### Real-Time Quantitative PCR

Total RNA was isolated and first-strand cDNAs were synthesized, respectively, using a total RNA isolation system and First-strand cDNA Synthesis Kit (Tiangen, Beijing, China). All qRT-PCR assays were used in a CFX96 Real-time system (BIO-RAD) according to manufacturer instructions. Three independent biological replicates were carried out for each sample. Primer sequences used for real-time quantitative PCR were described in **Supplementary Table S1**.

### Apple Calli Transformation

The ERF3 and MYC2 transgenic apple calli were obtained from Professor Hao’s laboratory (An et al., 2016; An et al., 2018). The constructed recombinant plasmids were introduced into *Agrobacterium tumefaciens* LBA4404. 15-day-old “Orin” apple calli were infected with *A. tumefaciens* for 20 min which were carrying recombinant plasmids, and the apple calli were cultured on agar solidified MS medium for 2 days at 24 °C in darkness. Then, the apple calli were transferred to selective medium containing 35 mg L^−1^ hygromycin and 300 mg L^−1^ carbenicillin.

### Dual-Luciferase Assay

Full-length *MdMYC2* and *MdERF3* sequences were amplified with the primers described in **Supplementary Table S1** and were inserted into pBI 121 vectors. The promoter of *MdAFS* (1,500 bp) was constructed in the pGreenII 0800-LUC vector. All constructs were individually transformed into *Agrobacterium* GV3101 and stored as glycerol stocks at -80°C. *Agrobacterium* cultures were prepared with infiltration buffer (10 mM MES, 10 mM MgCl_2_, 150 mM acetosyringone, pH 5.6) to an OD600 of 1.0. The mixtures of transcription factor and promoter were infiltrated into tobacco leaves by needleless syringes. A living imaging apparatus was used for luminescence detection. For each transcription factor-promoter interaction, at least three independent experiments were performed with four replicates in each experiment.

### Yeast One-Hybrid Assay

Y1H was used to detect verification of interaction between transcription factor and AFS as described by An et al. (2016). The MdMYC2 and MdERF3 gene was cloned into the pGADT7 vector and the promoter fragment of MdAFS were inserted into the pAbAi vector.

### Electrophoretic Mobility Shift Assays (EMSA)

EMSAs were performed as described previously (Li et al., 2017). The LightShift™ Chemiluminescent EMSA Kit (Thermo, USA) was used in EMSA experiment. Oligonucleotide probes were synthesized and labeled with biotin. Biotin-labeled probes were incubated with MdERF3-GST protein in a binding buffer for 25 min, and the free and bound DNAs were separated in an acrylamide gel.

### Transient Overexpression in Apple

Fruit injection assays were carried out as described previously (Li et al., 2012). The overexpression viral vectors MdMYC2-IL60-2 and MdMERF3-IL60-2 were generated and the mixed vectors were injected into the fruit peels. Two days after infiltration, the peel near the infiltration point was collected for volatile analysis.

### Volatile Compounds Analysis by GC-MS

Volatile analysis was carried out as in our previous study (Deng et al., 2015). The fresh apple peels were ground in liquid nitrogen and 0.3 g was extracted with 5 ml of extraction buffer in a sealed container. 10 μl of 3-Nonanone (0.4 g L^-1^) was an internal standard. The volatile compounds were collected by solid phase microextraction (SPME) and analyzed using GCMS-QP2010 with a FID detector (Shimadzu, Tokyo, Japan).

### Statistical Analysis

Statistical analysis of the data was performed with SPSS. Data points represent the mean values ± standard deviation of three biological replicates. Differences were considered statistically significant when * P < 0.05 and ** P < 0.01.

## Results

### JA and Ethephon Treatments Promote the Expression of MdAFS and Increase ɑ-Farnesene Content in Apple

**Figure 1:**
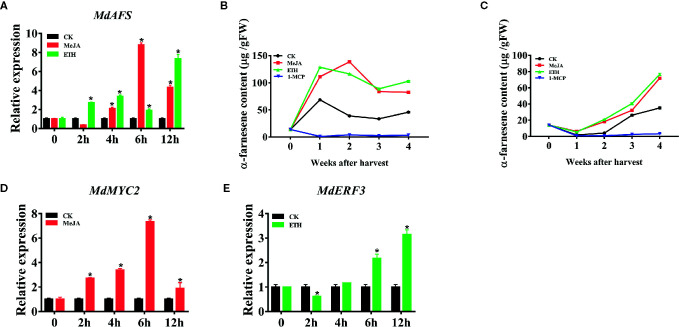
**(A)** Expression of the *MdAFS* gene in apple leaves under MeJA and ETH treatments. **(B, C)** Effects of MeJA, ETH and 1-MCP on production of α-farnesene. White Winter Pearmain apples were treated with JA, ETH, and 1-MCP, and stored at room temperature and 0℃ for 4 weeks to detect the content of α-farnesene. **(D, E)** Effect of MeJA and ETH treatments on *MdMYC2* and *MdERF3* expression in apple leaves. Data points represent the mean values ± standard deviation of three biological replicates * indicates P < 0.05 compared to CK.

In order to study the mechanism of regulation of α-farnesene synthesis, we first analyzed the promoter of *MdAFS*, which is the terminal enzyme gene in the α-farnesene synthesis pathway. In the *MdAFS* promoter, many potential *cis*-acting elements associated with hormone-related responses were identified, such as MeJA, Ethylene, and ABA (**Supplementary Table S2**). JA has been widely used in regulating plant growth and secondary metabolism, and significant progress has been achieved in regulating the accumulation of terpenoid secondary metabolites by its use. Ethylene is an important hormone and ethylene treatment reportedly involves in volatile synthesis and ethephon facilitates the release of the ethylene. To examine whether the *MdAFS* and α-farnesene were induced by phytohormones, “White winter pearmains” leaves and fruits were treated with MeJA and Ethephon. The results indicated that the expression of *MdAFS* was significantly upregulated after these treatments. When apple leaves were treated with MeJA, the maximum expression level of *MdAFS* occurred at 6 h post treatment (**Figure 1A**). The response of *MdAFS* to Ethephon treatment was peaked within 12 h post treatment (**Figure 1A**) and the α-farnesene content in the apple leaves increased with the increase of treatment time, reaching the highest in 12 h (**Supplementary Figure 1A**). Concomitantly, α-farnesene content was markedly higher than in controls following MeJA and Ethephon treatments, but markedly lower treated with 1-MCP relative (**Figures 1B, C**).

### Expression Profile of MdMYC2 and MdERF3 Correlated With MdAFS

It is reported that MYC2 and ERF play a significant roles in ethylene and JA signaling pathways. Combined with the previous promoter analysis and PlantCare software analysis results, we found that promoters contained MYCCONSENSUSAT (MYC), G-box, DRE and ERE motifs, which were reported to be the binding sites of MYC2 and ERF transcription factors, implying these transcription factors might involve in transcriptional regulation of MdAFS. Therefore, we selected transcription factors MdMYC2 and MdERF3 to test their correlation to MdAFS. As shown in **Figures 1D, E**, MdMYC2 and MdERF3 showed the same accumulation pattern as MdAFS in response to both MeJA and Ethephon treatments. The expression levels of MdMYC2 promoted at 2 h after MeJA treatment and peaked at 6 h; Further, the expression of MdERF3 peaked at 12 h. Gene expression analysis of *MdAFS*, *MdMYC2*, and *MdERF3* during room temperature storage of apple fruits under MeJA, ETH, and 1-MCP treatments showed the same results (**Supplementary Figures 1B–D**).

### MdMYC2 and MdERF3 Enhance the Transcription of MdAFS

**Figure 2:**
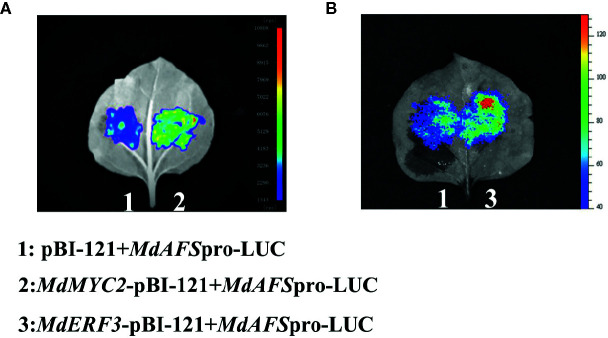
The fireﬂy luciferase (Luc) complementation imaging assays. *Agrobacterium* GV3101 strain harboring different constructs was infiltrated into tobacco leaves. Luminescence signals in the infiltrated region were measured 48 h after infiltration. **(A)** 1: pBI-121+MdAFSpro-LUC, 2: MdMYC2-pBI-121+MdAFSpro-LUC. **(B)** 1: pBI-121+MdAFSpro-LUC, 2: MdERF3-pBI-121+MdAFSpro-LUC.

**Figure 3:**
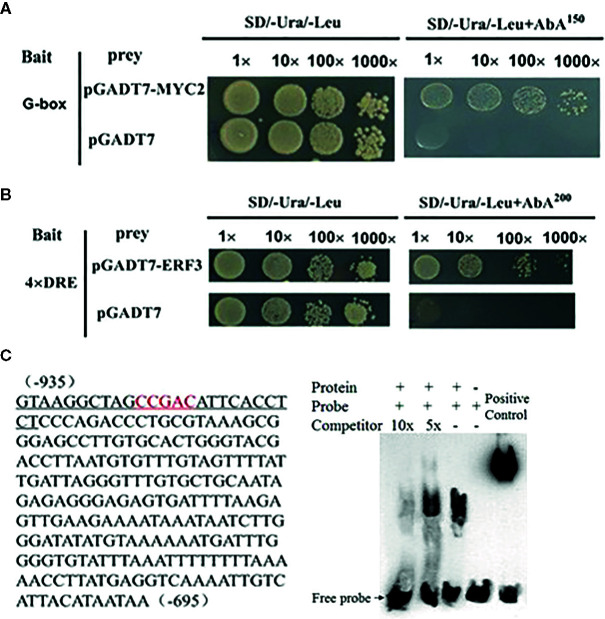
MdMYC2 and MdERF3 bind to the *MdAFS* promoter. **(A, B)** Yeast one-hybrid analysis of MdMYC2 and MdERF3 binding to *MdAFS* promoter. **(C)** EMSA indicating that MdERF3 directly bound to the *MdAFS* promoter at CCGAC *in vitro*. Biotin-labeled probes were incubated with MdERF3 protein and then separated in an acrylamide gel. The competitors were unlabeled probes.

We conducted a firefly luciferase (Luc) complementation imaging assay to test if MdMYC2 and MdERF3 could regulate the expression of *MdAFS*. As predicted, these two transcription factors showed trans-activation effects on the *MdAFS* promoter (**Figure 2**). Studies have shown that transcription factors MdMYC2 and MdERF3 can bind G-box and DRE elements to regulate the expression of downstream genes (Li et al., 2016; Li et al., 2017). Y1H assays were performed to test whether *MdMYC2* and *MdERF3* could bind promoters of *MdAFS*. Thus, G-box and four repeated DRE motifs were integrated into yeast cells. We found that, indeed, the two transcription factors were capable of binding both the G-box and the DRE motifs. In addition, to conform the binding results, we performed an EMSA with the MdERF3 together with 25 bp promoter fragments of *MdAFS* containing the DRE motif. The DRE motif of the *MdAFS* promoter was recognized by *MdERF3* (**Figure 3**). These results indicated that MdMYC2 and MdERF3 effectively activated the α-farnesene biosynthetic gene *MdAFS*, and MdERF3 directly bound the DRE motif in the *MdAFS* promoter.

### MdMYC2 and MdERF3 Overexpression Upregulate MdAFS in Stable Transgenic Apple Calli

**Figure 4:**
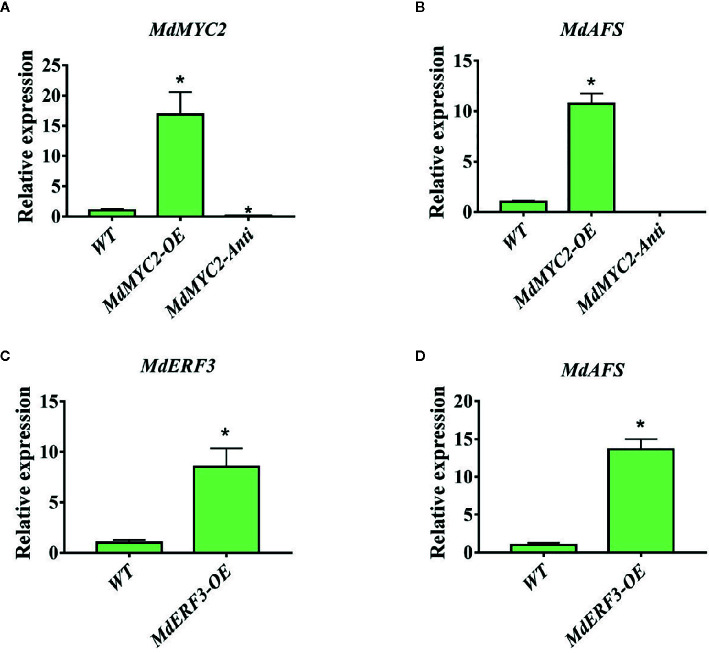
Expression analysis of *MdAFS*, *MdMYC2*, and *MdERF3* in transgenic apple calli. **(A)** Expression level of *MdMYC2* in apple calli overexpressing and silencing *MdMYC2.* **(B)** Expression level of *MdAFS* in apple calli overexpressing and silencing *MdMYC2.* **(C)** Expression level of *MdERF3* in apple calli overexpressing *MdERF3*. **(D)** Expression level of *MdAFS* in apple calli overexpressing *MdERF3.* Standard errors were calculated from three sets of biological replicates. * indicates P < 0.05 compared to WT.

To further examine the regulation of *MdAFS* by *MdMYC2* and *MdERF3*, overexpression and antisense constructs were transformed into “Orin” apple calli. The expression levels of *MdAFS* gene in the wildtype and transgenic calli were analyzed by qRT-PCR. As shown in **Figure 4**, we found that when *MdMYC2* expression was upregulated by ~15-fold, *MdAFS* expression was upregulated by ~10-fold in the calli overexpression *MdMYC2*. The expression levels of *MdAFS* were significantly lower in *MdMYC2*-antisense calli than in control calli. Similarly, *MdERF3*-overexpressing calli significantly upregulated expression of the *MdAFS* gene, which was increased over 13-fold. The α-farnesene content of calli were measured, and the results showed that the content of over-expressed *MdMYC2* and *MdERF3* calli were significantly higher than that of wildtype, and the α-farnesene content in antisense calli was the lowest (**Supplementary Figure 2**). These results showed that *MdMYC2* and *MdERF3* could positively regulate the expression of *MdAFS*.

### Transient Overexpression of MdMYC2 and MdERF3 in Apple Peels Increased the Production of α-Farnesene

**Figure 5:**
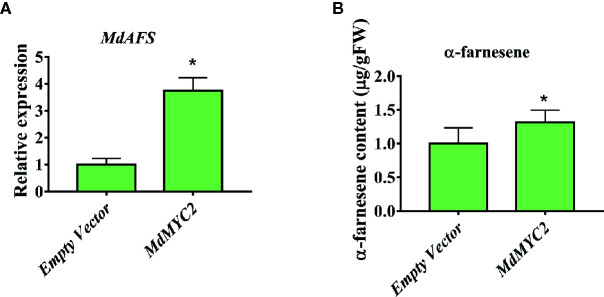
Transient expression of *MdMYC2* in apple peel. **(A)** Relative *MdAFS* gene expression in apple peel injected with Empty Vector and *MdMYC2*. **(B)** α-farnesene content in peel infiltrated with *MdMYC2*. More than three apples were injected for determination, and the data were the average value obtained. * indicates P < 0.05 compared to Empty Vector.

**Figure 6:**
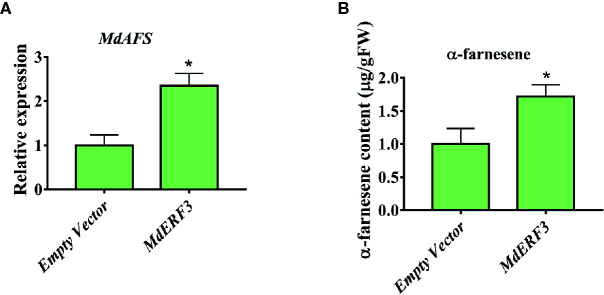
Transient expression of *MdERF3* in apple peel. **(A)** Relative *MdAFS* gene expression in apple peel injected with Empty Vector and MdERF3. **(B)** α-farnesene content in peel infiltrated with MdERF3. More than three apples were injected for determination, and the data were the average value obtained. * indicates P < 0.05 compared to Empty Vector.

There are technically and experimentally challenging in generation and testing of transgenic apple fruit (Kotoda et al., 2006). The assay was chosen to examine the role of *MdMYC2* and *MdERF3* in the biosynthesis of α-farnesene in apple fruits. The α-farnesene content of the peels infiltrated with *MdMYC2* was 8.97 μg g^–1^, respectively, representing a marked increase relative to the peels (7.4 μg g^–1^) infiltrated with empty vector (**Figure 5**). At the same time, apple peels infiltrated with *MdERF3* exhibited a significant increase by ~1.7-fold in the level of α-farnesene content (**Figure 6**).

### MdMYC2 and MdERF3 Affected Other Key Enzymes HMGR and FPPS in the Synthesis Pathway of α-Farnesene in Transgenic Apple Calli

**Figure 7:**
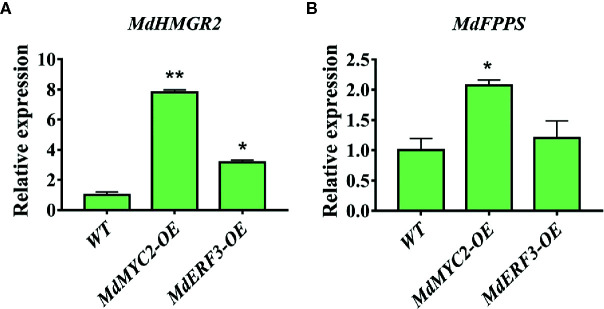
Expression analyses of *MdHMGR2* and *MdFPPS* in apple calli. **(A)** Expression analysis of *MdHMGR2* in overexpressing *MdMYC2* and *MdERF3* apple calli. **(B)** Expression analysis of *MdFPPS* in overexpressing *MdMYC2* and *MdERF3* apple calli. Each value is expressed as mean ± SE (n = 3). * indicates P < 0.05 compared to WT and ** indicates P < 0.01 compared to WT.

Transcription factors not only regulate single enzyme genes in a pathway, they also regulate the co-expression of multiple genes, thereby regulating the synthesis of specific secondary metabolites. It has been reported that the expression level of the *MdHMGR2* gene is positively correlated with α-farnesene and ethylene production in apples during low temperature storage (Rupasinghe et al., 2001). Nevertheless, studies on the *FPPS* gene in apples are scarce. Only two genes, *MdFPPS1* and *MdFPPS2*, have been cloned from apples (Yuan et al., 2013), and the similarity between them is as high as 99%. In addition, the regulation of *MdFPPS* on the synthesis of α-farnesene has not been clarified. However, our results demonstrated that overexpression of MdMYC2 and MdERF3 maybe affect the expression of the α-farnesene synthesis pathway genes *MdHMGR2* and *MdFPPS* (**Figure 7**).

## Discussion

Terpenes are the largest and most diverse class of chemicals among the volatile compounds produced by plants (Tholl, 2015). In addition to their phyto-ecological benefits, terpenes are also of great economic value to humans, as they can be widely used in flavors, agriculture and in the chemical industry (Bouvier et al., 2006). α-farnesene was first found in apple and was found to play a role in plant defense. Farnesene is the precursor of the biofuel farnesane, which has broad market value, as it has attracted extensive attention of the society in recent years (Peralta-Yahya et al., 2011). However, sesquiterpenes including α-farnesene are naturally produced in limited quantities (Wallaart et al., 1999). Therefore, metabolic engineering of organisms is an alternative and attractive way to produce these rare and valuable compounds.

Currently, heterologous production of α-farnesene can be carried out in the host of *Escherichia coli* and *Yarrowia lipolytica*. In *E. coli*, the heterologous expression of α-farnesene synthase from fruits made production of α-farnesene in bacterial (Zhu et al., 2014). Recently, the feasibility of producing α-farnesene in metabolically engineered *Y. lipolytica* was demonstrated for the first time (Yang et al., 2016).

In recent years, there have been many studies on transcriptional regulation, as the use of transcription factors is one of the effective methods to increase the yield of terpenoid secondary metabolites. Moreover, transcription factors can activate or inhibit the expression of several key enzymes in plant secondary metabolic biosynthetic pathways by interacting with cis-acting elements in target gene promoters, thus, they effectively start or close secondary metabolic biosynthetic pathways, and regulate the biosynthetic process of specific secondary metabolites, thereby effectively affecting their accumulation.

MYC transcription factors are the most widely separated and thoroughly studied bHLH transcription factors. Their conserved domains regulate the expression of target genes by combining with E-Box (CANNTG) or G-Box (CACGTG) elements of target promoters (Pires and Dolan, 2010). At the same time, MYC family members participate in plant growth and development, resistance to environmental stress, JA and other signal transduction processes, and also in the regulation of secondary metabolic pathways (Gao et al., 2015). MYC transcription factors have been isolated from species such as *Catharanthus roseus*, *Taxus*, *Artemisinin*, *Arabidopsis thaliana*, tomato, and apple. Among MYC transcription factors found in plants, MYC2 is the most in-depth studied that plays an important role in the JA-mediated signal regulation pathway in plants. MYC2 can enhance plant resistance to insects by positively regulating JA-induced insect-resistant genes. In *Arabidopsis thaliana*, MYC2 regulates JA-mediated resistance to insect pests, and tolerance to oxidative stress by enhancing ascorbic acid redox cycle and flavonoid biosynthesis (Dombrecht et al., 2007). In addition, MYC2 is a positive regulator of JAs-mediated secondary metabolite synthesis. In tobacco, NtMYC2a and NtMYC2b positively regulate the JA response gene PMT to promote nicotine formation (Zhang et al., 2012). In *Salvia sclarea*, WRKY and MYC2 transcription factors which are controlled by MJ elicitation, coactivate MEP-biosynthetic genes and accumulation of abietane diterpenes (Alfieri et al., 2018).

APETALA2/Ethylene (AP2/ERF) transcription factors play an important role in regulating plant growth, development and maturation, responses to biotic and abiotic stress, and secondary metabolism (Agarwal et al., 2006). The ERF subfamily responds primarily to abiotic stress either dependently or independently of plant hormones such as ethylene and various biotic stress phenomena, such as pathogens and insect attack (Lai et al., 2014). ERF transcription factors reportedly can bind specifically GCC-box or DRE/CRT elements. The terpenes can be regulated by transcription factors, including AP2/ERF. ZmERF58 is able to directly bind the promoter of *ZmTPS10* to synthesize E-β-farnesene and E-α-bergamot in maize (Li et al., 2015). *NtERF32* and related ERF genes are important non-*NIC2* locus related to transcriptional regulators of nicotine and total alkaloid formation (Sears et al., 2014). Further, ORA59, which is the AP2/ERF-domain transcription factor, and two GCC box binding sites that enables the *PDF1.2* gene to respond to the JA and ET signaling pathways (Zarei et al., 2011).

However, although there are several transcription factors being involved in terpene biosynthesis, the transcriptional regulation of the α-farnesene remains unclear. Similarly, it has been reported that jasmonic acid and ethylene, two important plant hormones, coordinate to regulate plant growth, development and tolerance to pathogens. Indeed, EIN3/EIL1, two important transcription factors in ethylene, reportedly mediate the signal interaction between jasmonic acid and ethylene; hence, EIN3/EIL1 is also a positive regulator of the jasmonic acid signaling pathway that regulates plant root development and resistance responses (Zhu et al., 2011). Thus, the question may be asked, is there a common transcription factor regulating α-farnesene synthesis in the JA and ET signaling pathways?

**Figure 8:**
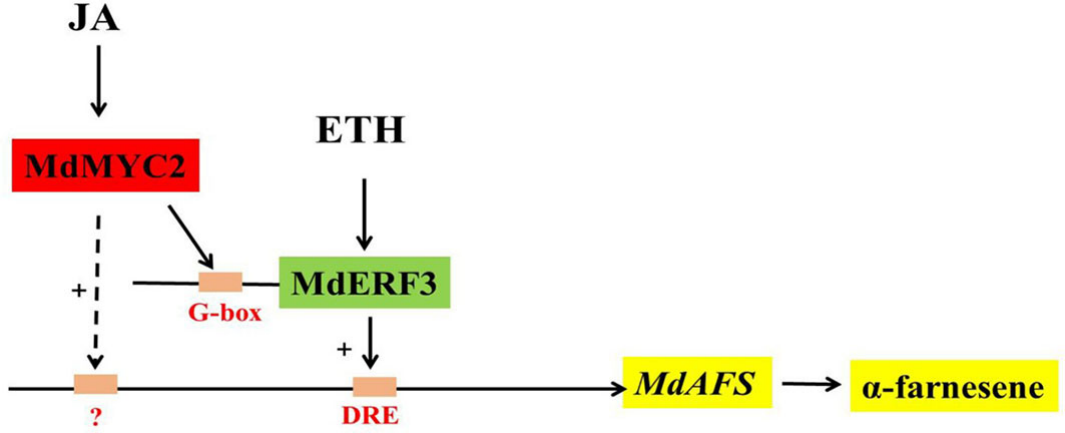
Model of synthesis of α-farnesene regulated by MdMYC2 and MdERF3.

In this study, we began with the regulation of these two hormones on α-farnesene. We found that whether stored at room temperature or at low temperature, JA and ET increased and 1-MCP decreased α-farnesene biosynthesis in apple fruit (**Figures 1B, C**). ERF and MYC2 are not only the key regulators in JA signaling pathway, but also the nodal factors connecting JA, ET, and other signal hormones. In addition, most steroid and alkaloid biosynthesis regulation is related to ERF and MYC2. Consistently with previous studies, we selected MdMYC2 and MdERF3 transcription factors for research, finding that these two transcription factors showed a similar expression pattern to *MdAFS* (**Figure 1**). This suggested that transcription factors MdMYC2 and MdERF3 might share a putative common regulatory mechanism of α-farnesene biosynthesis in plants. Further investigation of the transcriptional regulation of *MdAFS* gene by MdMYC2 and MdERF3, together with results of the dual-luciferase assay, the Y1H assay, and EMSA, led to the conclusion that MdMYC2 and MdERF3 is participated in transcriptional regulation of the *MdAFS* gene (**Figures 2** and **3**). In addition, to study the role of MdMYC2 and MdERF3, transient overexpression experiments were carried out. Apple peel infiltrated with MdMYC2 and MdERF3 showed a marked increase α-farnesene (**Figures 5** and **6**). Additionally, we found that transcription factors MdMYC2 and MdERF3 could regulate the expression of key enzyme genes *MdAFS*, *MdHMGR2*, and *MdFPPS* in the synthesis pathway of α-farnesene in stable transgenic apple calli (**Figures 4** and **7**). This indicated that MdMYC2 and MdERF3 effectively improved the synthesis of α-farnesene by activating the co-expression of multiple genes in the α-farnesene biosynthetic pathway. Consistently, it has been reported that MdMYC2 enhanced the transcription of MdERF3 by binding its promoter (Xu et al., 2017). We analyzed the transcript level of *MdERF3* in apple calli after overexpressing *MdMYC2*, and the results showed that the expression of *MdERF3* was increased in the overexpressing *MdMYC2* apple calli (**Supplementary Figure 3**). Although we failed to verify that MdMYC2 binds directly the promoter of gene *MdAFS*, our studies showed that transcription factor MdMYC2 promoted the synthesis of α-farnesene by regulating the expression of *MdAFS*. Based on these results and earlier studies, we propose the hypothetical working model shown in **Figure 8** to explain the synthesis of α-farnesene and the involvement of MdMYC2 and MdERF3 in its regulation.

It has been suggested that superficial scald development in apple and pear is related to α-farnesene and its oxidation products, namely, conjugated trienols (CTols), which accumulate progressively in the fruit peel during storage (Bordonaba et al., 2013). Previous investigations showed that l-MCP treatment induced scald resistance while inhibiting ethylene-dependent ripening. This indicated that the ethylene metabolic pathway may also be involved in the process of superficial scald (Karagiannis et al., 2018). Additionally, analysis of “Granny Smith” apples suggested that ethylene-related transcription factors might regulate *AFS* transcription during low temperature and then regulate the synthesis of α-farnesene and the occurrence of fruit superficial scald (Busatto et al., 2018). This study provided new insights into the regulation of α-farnesene and the mechanism of superficial scald.

In summary, this is the first report on the mechanism of regulation of the biosynthesis of α-farnesene at the transcriptional level. Our results indicate that MdMYC2 and MdERF3 exert their regulatory effects as positive regulators of α-farnesene biosynthesis-related genes. Further, our study identified key candidate genes and new strategies for using metabolic engineering methods to achieve high yields of α-farnesene.

## Data Availablity Statement

The datasets generated for this study are available on request to the corresponding authors.

## Author Contributions

Designed the experiments: QW, YH, and YZ. Performed the experiments: QW and HL. Analyzed the data: QW, HL, MZ, and SL. Wrote the paper: QW, HL.

## Funding

This study was financially supported by the National Natural Science Foundation of China (No. 31370359).

## Conflict of Interest

The authors declare that the research was conducted in the absence of any commercial or financial relationships that could be construed as a potential conflict of interest.

## Supplementary Material

Supplementary Figure 1
**(A)** α-farnesene content in apple leaves under MeJA and ETH treatments. **(B)** Expression of the MdAFS gene in apples stored at room temperature for 4 weeks under MeJA, ETH and 1-MCP treatments. **(C)** Expression of the MdMYC2 gene in apples stored at room temperature for 4 weeks under MeJA, ETH and 1-MCP treatments. **(D)** Expression of the MdERF3 gene in apples stored at room temperature for 4 weeks under MeJA, ETH, and 1-MCP treatments.

Supplementary Figure 2 α-farnesene content in apple calli overexpressing *MdMYC2*, *MdERF3* and silencing *MdMYC2*.

Supplementary Figure 3 Analysis of *MdERF3* transcript level in apple calli after overexpression *MdMYC2*.
