# Supplementary material for: MdMYC2 and MdERF3 Positively Co-Regulate α-Farnesene Biosynthesis in Apple
Source: Front Plant Sci. 2020 Sep 2;11:512844. doi: 10.3389/fpls.2020.512844 (PMC7492718; doi:10.3389/fpls.2020.512844)
Supplement: Supplementary file 1 [file Table_1.docx]

**Supplementary Table S1.** Primers used for gene expression analysis and vector construction in this study.

| Genes | Primers | Sequences (5′-3′) |
| --- | --- | --- |
| *MdAFS*pro | Forward | CATGCCGATCATCCAGCCA |
|  | Reversed | AGTGAATACCTAATTTGGTGTACAAG |
| *MdMYC2* | Forward | ATGACGGACTACCGGATAC |
|  | Reversed | AATCTGGCGGGCATCG |
| *MdERF3* | Forward | ATGTACGGACAGAGTGGAG |
|  | Reversed | GACCACCAATAACTGCTCGCCAC |
| qRTMdAFS | Forward | AAATCTGGGCTTCGCAGACA |
|  | Reversed | GCTCAGGCTCGAATGCTACT |
| qRTMdMYC2 | Forward | TTGGAGGTCGGTTCTTGGC |
|  | Reversed | GGGCTTGGGTTCTCGGATAAG |
| qRTMdERF3 | Forward | GCGAAGGGGAAGCATTACAG |
|  | Reversed | TCCCTGCTCCCATTTCCAAT |
| qRTMdHMGR2 | Forward | GGTTGCCAGCACCAATAGGG |
|  | Reversed | TGAACACGACAGCGAGGGAAT |
| qRTMdFPPS | Forward | AACGAGGTAGAGTTTCAAACAGC |
|  | Reversed | CGCAAGCAACAGGAAGATA |
| pAbAi-G-box | Forward | CCTATATAACCCACCCCTCCG |
|  | Reversed | AGTGAATACCTAATTTGGTGTACAAG |
| pAbAi-DRE | Forward | CTTGCTAGCCGACATGCTAGCCGACATGCTAGCC  GACATGCTAGCCGACATCTC  GAGATGTCGGCTAGCATGTCGGCTAGCATGTCGGC  TAGCATGTCGGCTAGCAAG |
|  | Reversed |  |
| EMSA-DRE | Forward | GTAAGGCTAGCCGACATTCACCTCT |
|  | Reversed | AGAGGTGAATGTCGGCTAGCCTTAC |
