# Supplementary material for: MdMYC2 and MdERF3 Positively Co-Regulate α-Farnesene Biosynthesis in Apple
Source: Front Plant Sci. 2020 Sep 2;11:512844. doi: 10.3389/fpls.2020.512844 (PMC7492718; doi:10.3389/fpls.2020.512844)
Supplement: Supplementary file 2 [file Table_2.docx]

**Supplementary Table S2.** Putative cis-acting elements in the *MdAFS* promoter.

| Regulatory element | Sequence | Location | Function |
| --- | --- | --- | --- |
| ABRE | ACGTG | 484(-) 958(-) 1891(+) | abscisic acid-responsive element |
| CGTCA-motif | CGTCA | 960(+) | methyl jasmonate-responsive element |
| DRE core | GCCGAC | 1098(+) | abiotic stress-responsive element |
| ERE | ATTTCAAA | 81(+) 1508(+) | ethylene-responsive element |
| MYB | CAACCA | 1546(-) | MYB binding sites |
| MYC | CATTTG,CAATTG,  CATGTG | 47(+) 304(+) 963(+) 1341(+)  1623(+) 1943(+) | MYC binding sites |
