# Supplementary figures and images for: MdMYC2 and MdERF3 Positively Co-Regulate α-Farnesene Biosynthesis in Apple
Source: Front Plant Sci. 2020 Sep 2;11:512844. doi: 10.3389/fpls.2020.512844 (PMC7492718; doi:10.3389/fpls.2020.512844)

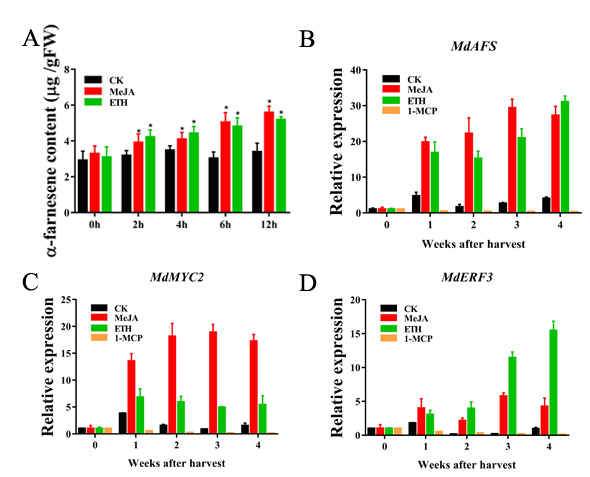

Supplement: Supplementary Figure 1 — (A) α-farnesene content in apple leaves under MeJA and ETH treatments. (B) Expression of the MdAFS gene in apples stored at room temperature for 4 weeks under MeJA, ETH and 1-MCP treatments. (C) Expression of the MdMYC2 gene in apples stored at room temperature for 4 weeks under MeJA, ETH and 1-MCP treatments. (D) Expression of the MdERF3 gene in apples stored at room temperature for 4 weeks under MeJA, ETH, and 1-MCP treatments. [file Image_1.jpeg]

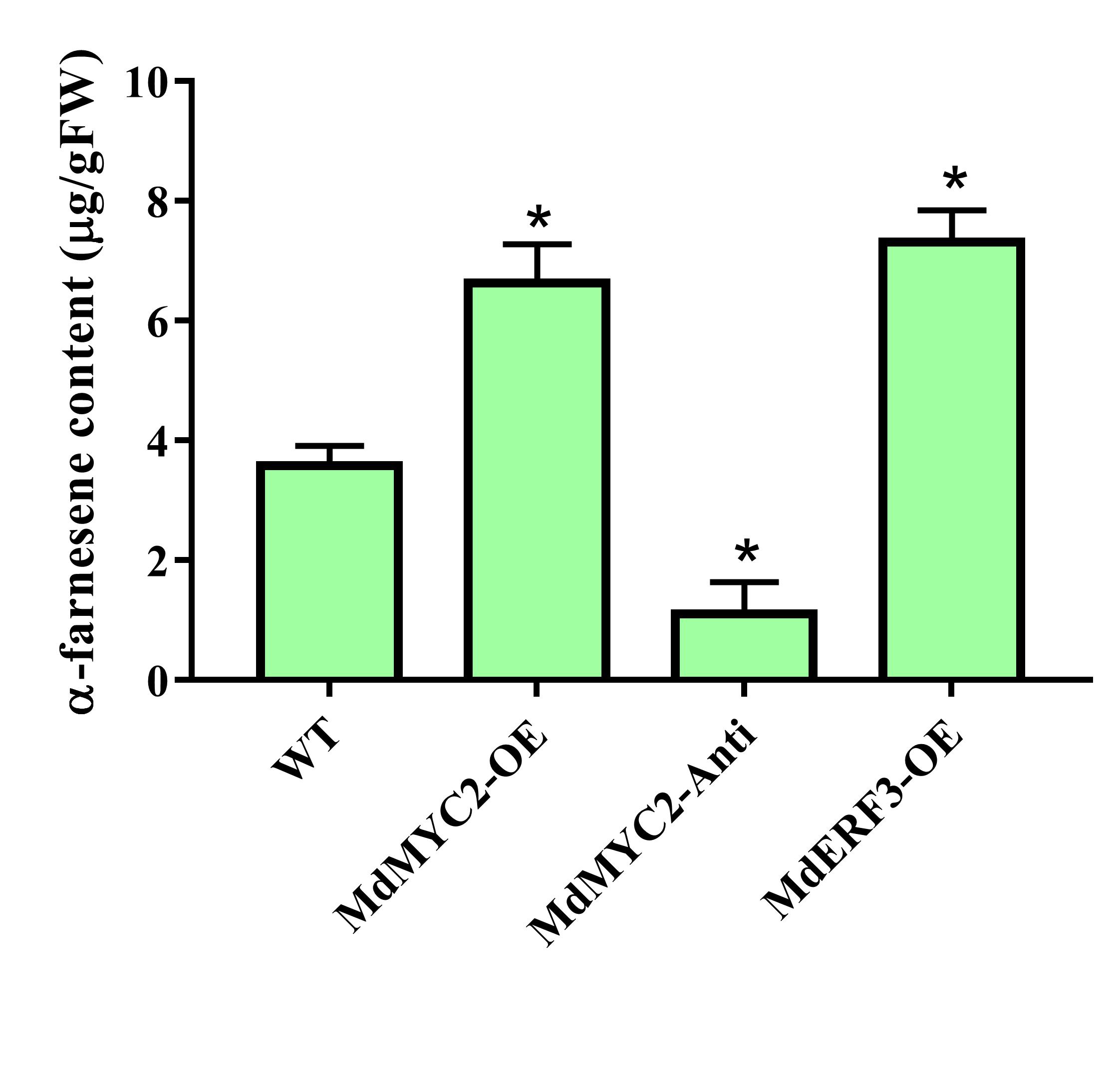

Supplement: Supplementary Figure 2 — α-farnesene content in apple calli overexpressing MdMYC2, MdERF3 and silencing MdMYC2. [file Image_2.jpeg]

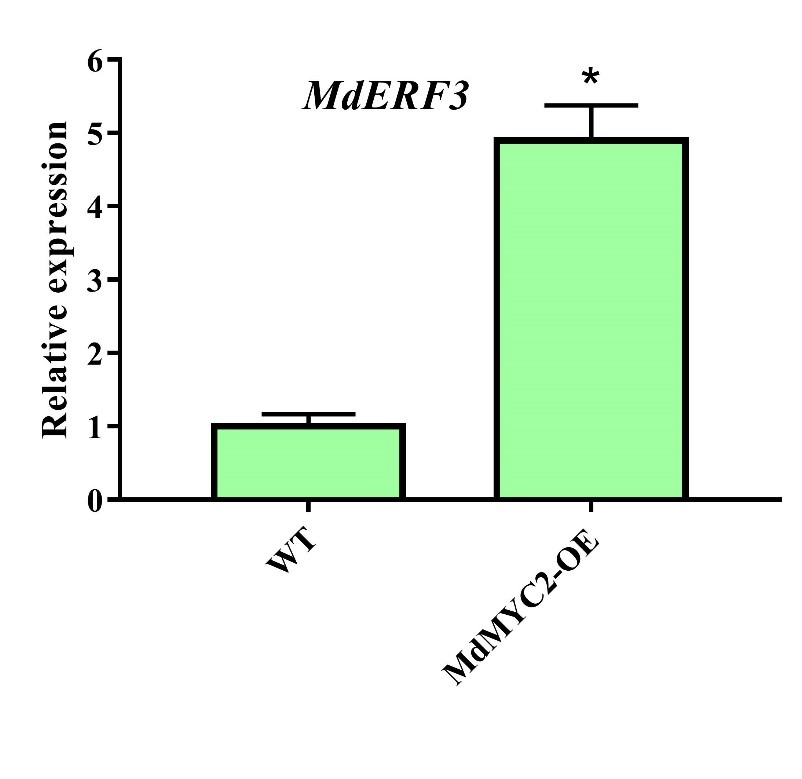

Supplement: Supplementary Figure 3 — Analysis of MdERF3 transcript level in apple calli after overexpression MdMYC2. [file Image_3.jpeg]
